# Long intergenic non-protein coding RNA 00707 regulates chondrocyte apoptosis and proliferation in osteoarthritis by serving as a sponge for microRNA-199-3p

**DOI:** 10.1080/21655979.2022.2061287

**Published:** 2022-04-29

**Authors:** Yan Xu, Liang Duan, Shizhang Liu, Yuanyuan Yang, Zhi Qiao, Liang Shi

**Affiliations:** a Department of Orthopedics, Xi’an Fifth Hospital, Xi’an, Shaanxi, PR China; b Department of Orthopedics, Shaanxi Provincial People’s Hospital, Xian City, Shaanxi, PR China; c Editorial Board of Chinese Journal of Child Health Care, the Second Affiliated Hospital of Xi ‘An Jiaotong University, Xian, Shaanxi, China

**Keywords:** LINC00707, miR-199-3p, proliferation, apoptosis, osteoarthritis

## Abstract

It is known that long intergenic non-protein coding RNA 00707 (LINC00707) promotes lipopolysaccharide (LPS)-injury and microRNA-199-3p (miR-199-3p) regulates chondrocyte proliferation and apoptosis. Both processes participate in osteoarthritis (OA). We predicted that LINC00707 and miR-199-3p may interact with each other. Therefore, LINC00707 and miR-199-3p may interact with each other to participate in OA. In this study, the expression of LINC00707 and miR-199-3p in both OA and normal articular cartilage tissues was analyzed using RT-qPCR. The subcellular location of LINC00707 and its direct interaction with miR-199-3p were explored by nuclear fractionation assay, RNA pull-down assay and Luciferase reporter assay, respectively. The role of LINC00707 and miR-199-3p in regulating the expression of each other was analyzed using an overexpression assay, followed by RT-qPCR. The role of LINC00707 and miR-199-3p in regulating OA chondrocyte proliferation and apoptosis was analyzed by BrdU assay and cell apoptosis assay, respectively. OA tissues exhibited increased expression of LINC00707 and decreased expression of miR-199-3p. LINC00707 directly interacted with miR-199-3p in cytoplasm. However, LINC00707 and miR-199-3p overexpression failed to affect each other’s expression. LPS treatment increased LINC00707 expression and decreased miR-199-3p expression in OA chondrocyte. LINC00707 overexpression increased the apoptosis of OA chondrocytes induced by LPS and suppressed the proliferation of OA chondrocytes. Moreover, LINC00707 suppressed the role of miR-199-3p in enhancing cell proliferation and suppressing cell apoptosis. In conclusion, LINC00707 can be detected in cytoplasm and it may sponge miR-199-3p to regulate chondrocyte proliferation and apoptosis in OA.

## Graphical Abstract

**Figure:**
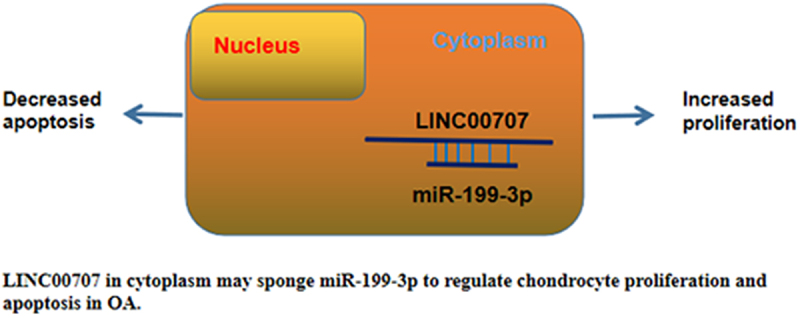

## Highlights

- LINC00707 directly interacted with miR-199-3p.
- LINC00707 overexpression suppressed the proliferation of osteoarthritis chondrocytes.
- LINC00707 suppressed the role of miR-199-3p in enhancing cell proliferation and suppressing cell apoptosis.

## Introduction

As the most common type of arthritis, osteoarthritis (OA) is caused by the progressive degenerative change in one or multiple knees, hands or hips cartilage [1]. OA mainly affects people at middle or old age [2]. In OA patients, the overgrowth and thickening of adjacent bones and the chronic inflammation may lead to deformation of joints, chronic pain and stiffness, seriously affecting patients’ life quality [3,4]. OA can usually be treated with anti-inflammatory medicines. However, no cure is available for OA, and the physiological changes in OA patients cannot be reversed [5]. In addition, the etiology of OA is still hardly known and is likely multifactorial, leading to difficulties in the development of treatment and preventative approaches [6,7].

Although advances have been made in the treatment of OA, such as the development of platelet-rich plasma and transplantation of mesenchymal stem cells [8,9], these novel therapies are either limited by the low therapeutic efficacy or are still under research [8,9]. Being an emerging anti-OA approach, targeted therapy focuses on the treatment of OA by regulating disease-related gene expression, such as Wnt signaling and TFEB gene expression [10,11]. However, effective targets remain lack.

Increasing evidences show that long noncoding RNAs (lncRNAs) play a vital role in cell biology processes, such as cell apoptosis and proliferation, cell differentiation, metastasis and tumorigenesis [12,13]. Moreover, abnormally expressed lncRNAs can lead to multiple abnormal expressions of genes associated with disease and biological functions [13]. For instance, lncRNA Metastasis-Related Lung Adenocarcinoma Transcript 1 could sponge for miR-150-5p and regulate osteoarthritis proliferation and apoptosis in OA [13]. OA is an inflammatory disease, in which, inflammatory factors, such as TNF-α, MAPK and MMP13 play critical roles [14–16]. It has been well established that lncRNAs are also critical players in inflammatory diseases [17–19]. Previous studies showed that long intergenic non-protein coding RNA 00707 (LINC00707, chromosome 10, 3087 nucleotides) knockdown accelerates osteogenic differentiation of human bone marrow‑derived mesenchymal stem cells by regulating DKK1 via targeting miR‑103a‑3p [20]. However, the function LINC00707 in OA has not been fully explored.

MicroRNAs (miRNAs) are small noncoding RNAs 22–24 nt in length [21], and are involved in the regulation of OA. A previous study indicated that microRNA-199-3p (miR-199-3p) overexpression facilitates chondrocyte proliferation and suppresses cell apoptosis in knee OA via DNMT3A repression [22]. We predicted that miR-199-3p may be able to bind to LINC00707. Therefore, a potential interaction between them may exist in OA. In the present study, we sought to explore the function of LINC00707 and its underlying mechanisms in OA. In addition, whether miR-199-3p is associated with the function of LINC00707 was also addressed.

## Materials and methods

### Articular cartilage tissues

To analyze differential gene expression in OA, articular cartilage tissues, which are directly involved in the pathogenesis of OA, were collected from 38 OA patients prior to the initiation of therapies. These patients underwent knee arthroplasty at Shaanxi Provincial People’s Hospital between March 2018 and January 2021. Ethics approval was obtained from the Ethics Committee of this hospital (Supplemental file 1). This study was carried out in accordance with the Declaration of Helsinki. To perform control experiments, control articular cartilage tissues were also collected from 38 non-OA patients who underwent total hip replacement surgery at the same hospital due to femoral neck fracture caused by accident. No pathological changes in control articular cartilage samples were observed. All patients signed informed consent. Please check Table 1 for clinical features of both groups of patients.

**Table 1.** Clinical features of OA and Control groups

	OA (*I* = 38)	Control (*n* = 38)
Gender (Female)	27	27
Age (Years, mean ± SD)	56.72 ± 7.49	56.23 ± 8.24
Obesity %	12	10
Disease duration (months ± SD)	62,12 ± 12.34	NA
Kellgren–Lawrence stage		
III	14	NA
IV	24	NA
Smokers (%)	16	17
Drinkers (%)	23	21

### Chondrocytes and cell culture

Human chondrocytes isolated from an adult with OA were purchased from Sigma-Aldrich (402OA-05A). DMEM containing 10% FBS (Gibco) was used to resuspend chondrocytes. Culture medium was also added with l-glutamine (2 mM) and penicillin/streptomycin (units). Cells were seeded on to a 6 cm plate with a cell density of 10^6^ cells per plate. Cells were first cultivated for 5 days, followed by a second cell culture at a cell density of 5 × 10^5^ cells per well. In cases of LPS treatment, cells were cultivated in a medium containing LPS at a density of 0, 2, 4, 6, 8 and 10 μg/ml for 48 h prior to use.

### Cell transfections

Chondrocytes were transfected with pcDNA3.1-LINC00707 vector or mimic of miR-199-3p (Invitrogen) through Lipofectamine 2000 (Invitrogen)-mediated transient transfections. All operations were completed following manufacturer’s instructions. Negative controls (NC) were included by performing NC miRNA or empty vector transfections. Overexpression was checked every 24 h until 72 h.

### RNA preparations and RT-qPCRs

Direct-zol RNA Kit (Zymo Research) was used to isolate total RNA from all samples. Treatment with DNase I (Sigma-Aldrich) was performed to completely remove genomic DNA. Bioanalyzer was then used to analyze RNA integrity to make sure a RIN higher than 8 was obtained in each case.

With 1000 ng total RNA as templet cDNA samples were prepared through reverse transcription performed using SSRT IV kit (Invitrogen), followed by qPCR performed using SYBR ® Green Quantitative RT-qPCR Kit (Sigma-Aldrich) to determine the expression of LINC00707 with 18S rRNA as internal control. Primer sequences were: 5'-TCACATCTGTGAAAAGAGTGC-3' (forward) and 5'-TGGACTGTGAGTACCAGGC-3' (reverse) for LINC00707; 5'-TCATAAGCTTGCGTTGATT-3' (forward) and 5'-AGTCAAGTTCGACCGTCTT-3' (reverse) for 18S rRNA. Hairpin-it TM miRNAs qPCR kit (Genepharma) was used to determine the expression of miR-199-3p with RNU6B internal control. The 2^−ΔΔCT^ method was used to normalize Ct values [23]. Values of ΔCT were first calculated and the one with the highest value of ΔCT was set to value ‘1’. All other samples were normalized to this sample.

### RNA pull down assay

Biotinylated miR-199-3p (Bio- miR-199-3p) and NC miRNA (Bio-NC) were both purchased from Invitrogen (Shanghai, China). Following cell transfections, cells were cultivated for 48 h, followed by incubation with cell lysis buffer on ice for 30 min to prepare cell lysate. After that, RNA-bound beads adhered streptomyces affidins were mixed with cell lysate to perform RNA pull-down. After that, RNA purification was performed. RT-qPCR was then carried to analyze the expression of LINC00707.

### Nuclear fractionation

Chondrocytes were used to prepare cytoplasm and nuclear samples with Cytoplasmic and Nuclear RNA Purification Kit (Norgen, Ontario, Canada). Two fractions were simply separated by centrifugation for 10 min at 2500 g. Following RNA isolation, PCR was performed with GAPDH as a cytoplasmic marker to determine the expression of LINC00707. PCR products were separated by 1.5% agarose gel and stained with EB. After that, MyECL imager was used to collect images

### BrdU incorporation assay *[24]*

DNA synthesis, which is directly related to BrdU incorporation, was used to reflect cell proliferation. Cells harvested at 48 h post-transfection were seeded on to a 96-well cell plate with a density of 10^3^ cells per well. Three replicate cases were set for each experiment. BrdU (BD Pharmingen) was added to final concentration of 10 μM, followed by incubation for 2 h. After medium removal, cell fixation was performed for 1 h and incubation with peroxidase-coupled anti-BrdU-antibody (Sigma-Aldrich) for 2 h was done, followed by PBS washing for 3 times. Incubation with tetramethylbenzidine was performed for 0.5 h and OD values were determined at 450 nm. Cells incubated with BrdU but its antibody was not added.

### Cell apoptosis assay

Serum-free medium was added with 10 μg/ml LPS to culture chondrocytes for 24 h. Three replicate cases were set for each experiment, in each replicate 10^3^ cells were included and cultivated in a 96-well cell plate. Following digestion with 0.25% trypsin, Annexin V-fluorescein isothiocyanate (FITC) and propidium iodide (PI) staining was performed for 2 h in dark. After washing with PBS, cell apoptosis was then analyzed with flow cytometry. Annexin V-FITC+/PI- and % Annexin V-FITC+/PI+ cells were calculated (apoptotic rate).

### Luciferase reporter assay *[25]*

Full length LINC00707 was cloned into pGL3 plasmids (Promega) to establish the luciferase vector of LINC00707. Chondrocytes were transfected with LINC00707+ miR-199-3p or LINC00707+ miRNA NC. After transfection cell culture was performed under the aforementioned methods. Luciferase activity was detected by Dual Luciferase Reporter Assay Kit (Promega Corporation) using cells harvested at 24 h post-transfection.

### Statistical analysis

Two independent group comparisons were performed with the unpaired *t* test. ANOVA (one-way or two-way) Tukey’s test was used for comparisons of more than 2 independent groups. *P* < 0.05 was statistically significant.

## Results

### RT-qPCR analysis of the expression of LINC00707 and miR-199-3p in OA

**Figure 1.**
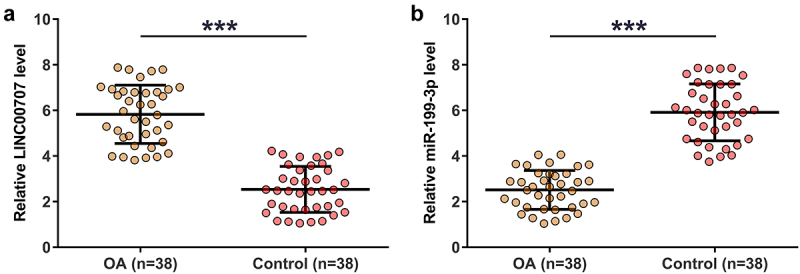
RT-qPCR analysis of the expression of LINC00707 and miR-199-3p in OA.

Gene expression determines function, therefore gene expression analysis is critical for functional characterization. In this study, the 38 control and OA articular cartilage tissues were used to analyze the differential expression of LINC00707 and miR-199-3p through RNA isolation and RT-qPCR. Compared to control samples, OA tissues exhibited increased expression of LINC00707 (Figure 1(a), *p* < 0.001) and decreased expression of miR-199-3p (Figure 1(b), *p* < 0.001). Our data illustrated the potential involvement of LINC00707 and miR-199-3p in OA.

### Subcellular location of LINC00707 in chondrocytes and analysis of its interaction with miR-199-3p

**Figure 2.**
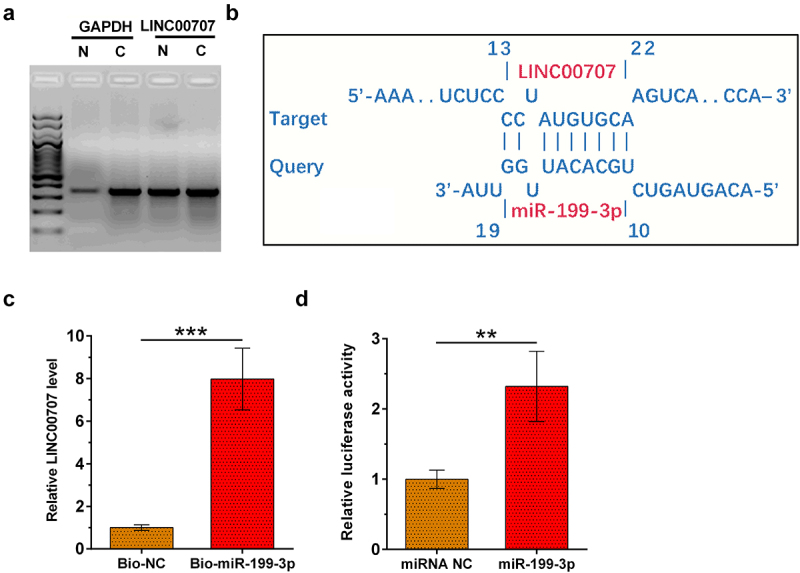
Subcellular location of LINC00707 in chondrocytes and analysis of its interaction with miR-199-3p. To analyze the subcellular location of LINC00707, nuclear fractionation assay was performed on chondrocytes (a). The interaction between LINC00707 and miR-199-3p was predicted by IntaRNA 2.0 (b). The direct interaction between LINC00707 and miR-199-3p was analyzed by RNA pull-down assay (c). Dual-luciferase reporter assay was performed by transfecting LINC00707 and miRNA NC or LINC00707 and miR-199-3p into chondrocytes (d). *p < 0.05; ***p < 0.001.

Analysis of the subcellular location of a gene may provide insights to its function. To analyze the subcellular location of LINC00707, nuclear fractionation assay was performed on chondrocytes. As revealed by RT-PCR analyis, LINC00707 can be detected in both nuclear (N) and cytoplasm (C) samples. In contrast, expression level of GAPDH (cytoplasmic marker) is much higher in C sample than in N samples (Figure 2(a)). The interaction between LINC00707 and miR-199-3p was predicted by IntaRNA 2.0, which illustrated multiple potential base pairs between them (Figure 2(b)). The direct interaction between LINC00707 and miR-199-3p was analyzed by RNA pull-down assay. Compared to bio-NC group, Bio-miR-199-3p group exhibited significantly higher level of LINC00707 (Figure 2(c), *p* < 0.001). Dual-luciferase reporter assay was utilized to further analyze the interaction between LINC00707 and miR-199-3p. Compared to cells transfected with LINC00707 and miRNA NC, cells transfected with LINC00707 and miR-199-3p mimic showed significantly reduced relative luciferase activity (Figure 2(d), *p* < 0.05). Thus, LINC00707 may may directly interact with miR-199-3p in cytoplasm.

### Analysis of the regulator roles of LINC00707 and miR-199-3p in the expression of each other

**Figure 3.**
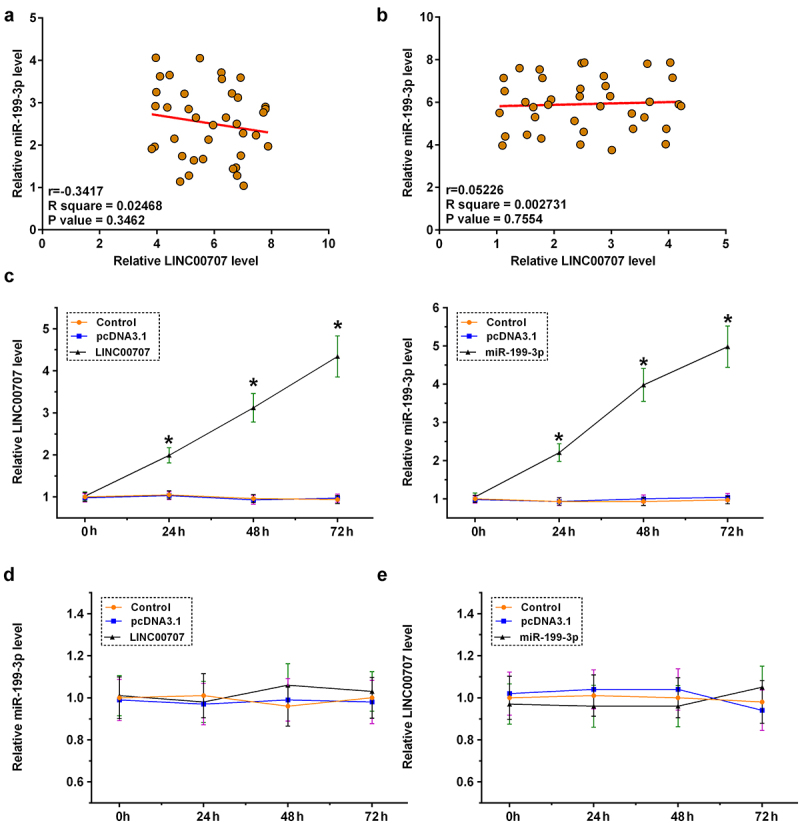
Analysis of the regulator roles of LINC00707 and miR-199-3p in the expression of each other. The direct interaction may indicate the regulator roles of LINC00707 and miR-199-3p in the expression of each other. Pearson’s correlation coefficient analysis was done to analyze the correlations between LINC00707 and miR-199-3p across OA (a) and control (b) samples. Chondrocytes were overexpressed with LINC00707 or miR-199-3p, and the overexpression was confirmed by RT-qPCR every 24 h until 72 h (c). The role of LINC00707 in the expression of miR-199-3p (d), and the role of miR-199-3p in the expression of LINC00707 (e) were analyzed by RT-qPCR. *p < 0.05.

The direct interaction between LINC00707 and miR-199-3p may indicate the regulator roles of LINC00707 and miR-199-3p in the expression of each other. Pearson’s correlation coefficient analysis showed that LINC00707 and miR-199-3p were not significantly correlated across OA (Figure 3(a)) and control (Figure 3(b)) samples. Chondrocytes were overexpressed with LINC00707 or miR-199-3p, and the overexpression was confirmed by RT-qPCR every 24 h until 72 h (Figure 3(c), *p* < 0.05). Interestingly, LINC00707 failed to affect the expression of miR-199-3p (Figure 3(d)), and the role of miR-199-3p in the expression of LINC00707 was also not significant (Figure 3(e)). Therefore, LINC00707 is unlikely a target of miR-199-3p and other interactions should be explored. Another possibility is that LINC00707 may serve as a sponge of miR-199-3p.

### Analysis of the roles of LINC00707 and miR-199-3p in the proliferation and apoptosis of chondrocytes

**Figure 4.**
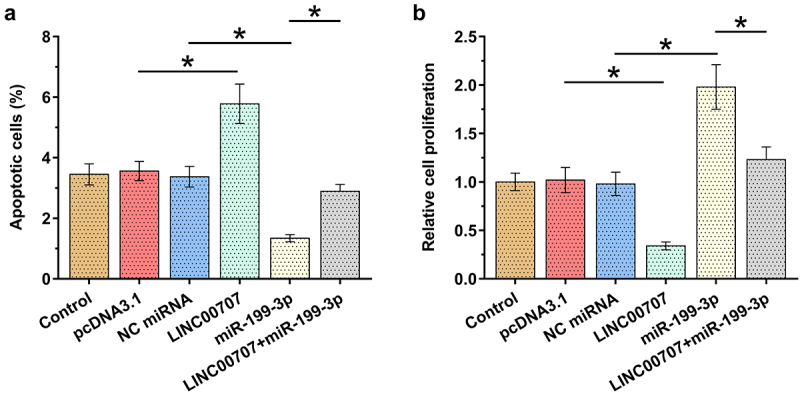
Analysis of the roles of LINC00707 and miR-199-3p in the proliferation and apoptosis of chondrocytes. The roles of LINC00707 and miR-199-3p in the proliferation and apoptosis (induced by 10 μg/ml LPS for 24 h) of chondrocytes were analyzed by BrdU assay (a) and cell apoptosis assay (b), respectively. *p < 0.05.

Alterations in proliferation and apoptosis of chondrocytes determine the progression of OA. To this end, the role of LINC00707 and miR-199-3p in the proliferation and apoptosis (induced by 10 μg/ml LPS for 24 h) of chondrocytes was analyzed by performing BrdU assay and cell apoptosis assay, respectively. LINC00707 overexpression increased the apoptosis of OA chondrocytes induced by LPS (Figure 4(a), *p* < 0.05) and suppressed the proliferation of OA chondrocytes (Figure 4(b), *p* < 0.05). Moreover, LINC00707 suppressed the role of miR-199-3p in enhancing cell proliferation and suppressing cell apoptosis. Therefore, LINC00707 may sponge miR-199-3p to suppress its role in cell proliferation and apoptosis. It is worth noting that LINC00707 and miR-199-3p failed to affect cell apoptosis under IL-1β and TNF-α treatment (data not shown).

## Discussion

Non-coding RNAs (ncRNAs) are emerging critical regulators in human diseases. This study mainly explored the crosstalk between two ncRNAs, namely, LINC00707 and miR-199-3p in OA. We found that LINC00707 and miR-199-3p expression was altered in OA. Additionally, LINC00707 may sponge miR-199-3p to regulate the proliferation and apoptosis of chondrocytes.

Zou et al. [23] explored the role of LINC00707 in injuries of MRC-5 cells induced by LPS. They found that suppressing the expression of LINC00707 could sponge miR-223-5p to reduce LPS-induced injury in MRC-5 [26]. It has been well established that LPS-induced inflammation participates in multiple human diseases, including OA [27], suggesting the involvement of LINC00707 in OA. Our study revealed the increased expression of LINC00707 in OA. As the only cells in healthy cartilage, chondrocytes produce collagen and proteoglycans to generate and maintain cartilaginous matrix [28]. The increased apoptosis and suppressed proliferation of chondrocytes not only participate in the initiation of OA, but also contributes to its progression [28]. This study showed that LINC00707 overexpression increased the apoptosis of OA chondrocytes induced by LPS and suppressed the proliferation of OA chondrocytes. Therefore, the overexpression of LINC00707 may promote OA initiation and progression by promoting the apoptosis of chondrocytes induced by LPS and suppressing cell proliferation.

We then explored the mechanism that mediates the function of LINC00707 in OA. DNMT3A contributes to OA progression [29]. MiR-199-3p targets DNMT3A to inhibit the apoptosis and enhance the proliferation of chondrocytes in knee OA [29]. Our study showed a decreased expression of miR-199-3p in OA and confirmed its regulatory role in the apoptosis and proliferation of chondrocytes. Interestingly, although LINC00707 and miR-199-3p directly interacted with each other, they failed to regulate the expression of each other. Additionally, the role of miR-199-3p in the apoptosis and proliferation of chondrocytes was suppressed by LINC00707. Considering the fact that mature miRNAs are only detected in cytoplasm and LINC00707 in this study was detected in both nuclear and cytoplasm, we speculate that LINC00707 in cytoplasm may sponge miR-199-3p to participate in OA. This is supported by correlation analysis, which revealed no close correlation between them across clinical samples. The function of an RNA sponge (or endogenous competing RNA) is to suppress the function of miRNAs without affecting its expression. In this study, miRNA expression was detected using RT-qPCR, in which high-temperature incubation was included and miR-199-3p can be released from LINC00707. Therefore, no close correlation was observed.

Besides our research, previous studies also reported the critcial function of several lncRNAs in OA. With an increased understanding of the role of lncRNAs in OA [30–32], lncRNAs are expected to be potential targets to treat OA.

## Conclusion

LINC00707 is overexpressed in OA and miR-199-3p is under-expression in OA. Additionally, LINC00707 may sponge miR-199-3p to regulate the proliferation and apoptosis of chondrocytes.

## Data Availability

The datasets generated and/or analyzed during the current study are available in https://115.com/s/swnywtq3zyl?password=u576&#, the code is u576.
